# Constant stress arches and their design space

**DOI:** 10.1098/rspa.2021.0428

**Published:** 2022-01

**Authors:** Wanda J. Lewis

**Affiliations:** School of Engineering, University of Warwick, Coventry CV4 7AL, UK

**Keywords:** constant stress arch, design space, moment-less arch, form-finding

## Abstract

It is generally accepted that an optimal arch has a funicular (moment-less) form and least weight. However, the feature of least weight restricts the design options and raises the question of durability of such structures. This study, building on the analytical form-finding approach presented in Lewis (2016. *Proc. R. Soc. A* **472**, 20160019. (doi:10.1098/rspa.2016.0019)), proposes constant axial stress as a design criterion for smooth, two-pin arches that are moment-less under permanent (statistically prevalent) load. This approach ensures that no part of the structure becomes over-stressed under variable load (wind, snow and/or moving objects), relative to its other parts—a phenomenon observed in natural structures, such as trees, bones, shells. The theory considers a general case of an asymmetric arch, deriving the equation of its centre-line profile, horizontal reactions and varying cross-section area. The analysis of symmetric arches follows, and includes a solution for structures of least weight by supplying an equation for a volume-minimizing, span/rise ratio. The paper proposes a new concept, that of a design space controlled by two non-dimensional input parameters; their theoretical and practical limits define the existence of constant axial stress arches. It is shown that, for stand-alone arches, the design space reduces to a constraint relationship between constant stress and span/rise ratio.

## 1. Introduction

### (a) Background and scope

The analysis presented here is inspired by the observation that the principle of constant stress governs the formation of natural objects, such as trees, bones or shells, all of which exhibit a minimal stress response to loading. This has implications for their durability and economy of material usage. These natural structures produce adaptive growth to maintain the state of constant stress [1]. Otto dedicated his life to studying natural forms and developing experimental form-finding methods that shaped structures using forces applied to them [2]. The extensive research programme completed by him and his team demonstrated that form-found structures are optimal in terms of their function, economy of material and aesthetic quality. The overall conclusion was that the path to defining optimal structures had its roots in nature. Alongside the experimental form-finding work, computational form-finding methods predicting shapes of a variety of structures were developed, as described in [3].

This paper builds on the contribution made in [4] describing two-pin moment-less arch forms of constant cross section, by proposing an alternative solution, in the form of moment-less arches of constant axial stress and, therefore, varied cross section. The principle of constant stress ensures that no part of the structure gets over-stressed under variable load (wind, snow and/or moving objects) relative to its other parts—a phenomenon observed in natural objects, such as trees, bones or shells. As explained in the opening paragraph, these objects are known to be highly optimized. The arches are subjected to permanent (statistically prevalent) load, the significance of which is discussed in §1c. The proposed theoretical model is based on the common assumption, used in [4] and in structural optimization research, that the structure is inextensible (rigid). This assumption has been tested on a linear elastic model of a moment-less arch, presented in [5] as a follow-on study from Lewis [4]. The results showed that, in moment-less arches of constant cross section (and, consequently, varied axial stress), the elastic effects, resulting in small deformations and bending action, were negligible. The arches were subjected to ultimate loads (comprising permanent + variable patch loads), and the moment-less forms exhibited a reduced stress response, compared with conventional arch forms of parabolic and circular configurations.

Arches discussed here are referred to as ‘fully loaded’—when carrying the deck and arch weight, or ‘stand-alone’—when carrying arch weight only. It is shown that stand-alone arches require separate attention, as they are subjected to different design constraints.

The theory considers the general case of an asymmetric arch configuration, from which a symmetric form is deduced. Asymmetry is produced by having arch supports placed at different levels. However, the bulk of the paper is devoted to symmetric forms to enable meaningful comparisons with previous work. It is shown that arches of least weight, which tend to be the main objective of structural optimization, are just a special case of constant stress, moment-less arch forms. The proposed analytical form-finding approach allows the shape of least weight structures to be found, by providing an explicit solution for the span/rise ratio, minimizing the arch volume.

The proposed form-finding approach makes a clear distinction between the independent input variables: span, rise, loading and constant value of stress, and the dependent output variables: horizontal reactions, centre-line profile, offset from the centre (in the case of an asymmetric arch) and material distribution in the structure.

The main advantage of an analytical approach over a computational one is its ability to generate a family of moment-less arches just by changing a few input parameters. It also produces smooth structural profiles—a feature that, in a computational model, would require a high level of discretization, adding to computational effort. In view of this, and with the plethora of papers published on optimal arch structures, the literature review presented below is limited to analytical studies.

### (b) Past work

The work on constant stress arches presented here has interesting parallels in other fields of study, which, broadly, fall into two diverse areas: suspension bridges and structural optimization.

#### (i) Suspension bridge research

Early work on suspension bridges generated a lot of interest in the shape of suspension chains supporting the structure. In the seventeenth century, it became known that the shape of a funicular (moment-less) arch was that of an ‘inverted suspension chain’—an analogy first demonstrated by Robert Hooke. His demonstration was limited to showing that the curve created by a hanging chain, when inverted, gives a suitable shape of a free-standing, constant cross-section arch, supporting its own weight. The mathematical description of the curve came later, referring to it as a catenary, expressed by a hyperbolic cosine function. The theoretical work that followed focused on the derivation of a curve adopted by a suspension chain working in constant stress under its own weight. The first contribution in this area was due to Gilbert [6] who described the curve as a ‘catenary of equal strength’. His derivation, given in Newton's fluxional calculus notation, and characterized by many divergences from contemporary terminology, was made accessible by Calladine [7]. A more concise form of the ‘catenary of equal strength’ equation, given in Cartesian coordinates, was later derived by Routh [8]. A more recent exposition of the role of the inverted suspension chain (or cable) analogy in describing moment-less forms of stand-alone arches is given in [3] and [9]. The analysis presented in [3] includes an example of the iconic Gateway Arch in St. Louis, the shape of which was described by the designer, Eero Saarinen, as ‘weighted catenary’, due to the varying cross section of the structure.

Early work on the ‘catenary of equal strength’ [6,8] was of limited value to the design of suspension and arch bridges, as it did not consider the presence of the deck weight. As far as the suspension bridges are concerned, this issue was addressed by Moseley [10] who reformulated the hanging chain equation for the combined chain and deck weight and called it ‘equation to the suspension chain of uniform strength’. Although the solution was somewhat convoluted, with the chain configuration expressed in terms of the varying tension force in the structure, the inverse form of the equation for the centre-line profile matched that of a constant stress arch. However, the work had a limited relevance to moment-less arch structures, as the span/rise ratio—one of the key factors defining their design space—lies outside that used in suspension bridges. Furthermore, with only a symmetric case being considered, it was not possible to deduce an asymmetric form. Finally, in common with the ‘catenary of equal strength’ and other approaches, the work used a permissible stress value, corresponding to an ultimate, rather than permanent, load—a point discussed in §1c.

#### (ii) Structural optimization research

Within the discipline of structural optimization, there is a considerable amount of literature dedicated to optimal arches, referred to as moment-less (funicular) structures of least weight. The concept of a least weight structure dates back to the nineteenth century and the work of Levy [11]. Using graphical methods, he researched bar frameworks and parallel cord trusses to determine which form was conducive to least weight. Later research by Rozvany & Wang [12], still focusing on optimal structural layouts of trusses and frames, drew on the work of Maxwell [13] and Mitchell [14]. After that, the research progressed to weight/cost minimization of arch and cable structures [15]. Hill *et al.* [16] derived profiles of ‘fully stressed’ arches, minimizing the total weight of the arch subject to the constant uniform (permissible) stress along its entire length. The work showed that the centre-line profile of the arch is unaffected by the external uniformly distributed load (deck weight). Building on plane Prager-structures research [12], Wang & Rozvany [17] derived centre-line profiles of a number of funicular arches of least weight, including asymmetric and symmetric forms under a uniformly distributed external load and arch weight. Focusing just on structural layouts, the work did not produce an expression for the cross-section area of the arch, which made the definition of the structure incomplete from a design point of view.

Unlike analytical form-finding, which finds a horizontal reaction from end conditions applied to the equation for the centre-line profile, structural optimization finds it from minimization of a cost/weight functional, to satisfy the condition of least weight. The approach cannot be applied to stand-alone arches, as the horizontal reaction does not affect their centre-line profile and there is no scope for volume/weight minimization.

As noted by Wang & Wang [18], the work of Marano *et al.* [19] on providing an optimal arch shape, reproduced the same arch profile as given in [16]; however, it did provide an expression for the varying cross-section area of the arch, thus providing more complete information, from a design point of view.

### (c) Significance of the proposed work

The approach presented here challenges the view that an arch of least weight constitutes the optimal design solution. To counter that, it proposes a broader concept, that of a constant stress arch, defined by analytical form-finding. Unlike approaches discussed above, the work addresses a number of design aspects, at the forefront of which is the question of the existence of constant stress arches. This has led to proposing a new concept, that of a Design Space. In the case of fully loaded arches, the design space is defined as a set of allowable combinations of two dimensionless input parameters, with their theoretical and practical limits clearly defined. In the case of stand-alone arches, the design space reduces to a constraint relationship between the value of constant stress and span/rise ratio.

Although the feature of least weight may appear attractive from the point of view of economy of material usage, at the same time, it restricts design options, by returning just one ‘optimal’ span/rise ratio as an outcome. In practice, factors such as the span and span/rise ratio are dictated by the environmental conditions of a prospective building site, as well as architectural and functional requirements, such as a minimum clearance/headroom. It is, therefore, an open question as to whether a least weight structure would readily satisfy all practical requirements. By using span/rise ratios as input variables, the analytical form-finding methodology implementing the principle of constant stress, opens up many creative opportunities within the design space; it allows not one, but a family of arches to be created. Further, it allows structures of least weight to be found from a volume-minimizing span/rise ratio, derived for this purpose (§4c). This demonstrates that arches of least weight are a special case of constant stress arches.

Having different objectives, the structural optimization and analytical form-finding treat the horizontal reaction differently, as discussed in §1b (ii).

While minimization of material weight/cost is the prime objective of structural optimization, in practice, such a cost is just a fraction of the overall design and construction budget. It is also worth noting that there is a potential trade-off between minimum weight and durability of the arch. Since it is not possible to produce an arch shape optimized for every conceivable load, it is sensible to select permanent load (assumed to be statistically prevalent), such as self-weight. This will ensure that the structure will work in an optimum state of uniform stress most of the time, and lead to enhanced durability. If, under ultimate load (comprising self-weight and live load combinations), the structure were to exceed its design strength, different values of input parameters, such as a lower value of constant stress, can be used to re-calculate the arch geometry. This forms a part of a usual, iterative design process.

In all the reviewed works, the use of the ultimate, not permanent, load was suggested in conjunction with permissible stress based on the elastic limit for the material strength. In contemporary structural design, permissible stress has been replaced by design strength, based on the probability that this value would not be exceeded during the lifespan of the structure. As both the applied load and the level of constant stress affect the geometry of the arch (its centre-line profile and cross-section area), using seemingly the same form of equation for the centre-line profile, but with different values of stress and loading, would produce different outcomes.

Objects shaped by the principle of constant stress can be found both in animate and inanimate nature. This study comments on an interesting parallel between a constant stress arch, and a (minimal) constant stress surface structure, exemplified by a soap film [3], as they are both subjected to limits defining their existence [20,21].

## 2. Analytical form finding: asymmetric form of a constant stress arch subjected to permanent load (arch self-weight and deck weight)

### (a) Equilibrium equations

**Figure 1.**
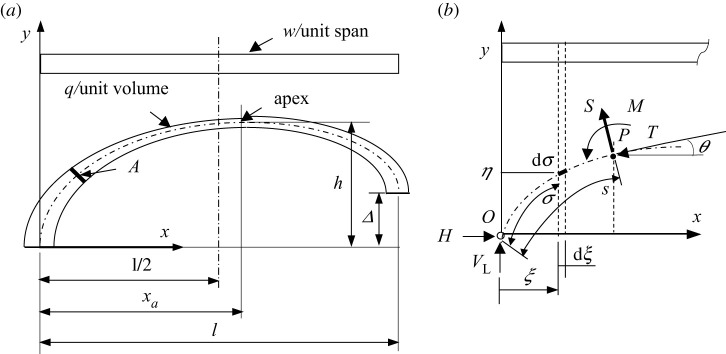
(*a*) Arch geometry and loading. (*b*) Arch centre-line profile, with its external and internal forces acting on the arch segment *OP*.

Figure 1*a* shows a general configuration of an asymmetrical arch of span, *l*, with one of the supports raised vertically by a distance, Δ. The total arch length, measured along its centre-line profile, is *C*, and its rise, *h*, appears not at mid-span, but a distance *x_a_* from the left support. The applied load consists of the deck weight, *w*, per unit span, and the arch self-weight represented by the volumetric weight density, *q*. In order to accommodate the requirement of constant stress, *f*, the arch has a varied cross-section area, *A*, along its length.

The arch is smooth and, at the apex, its cross-section area is *A*_0_. The deck can be supported by open spandrel columns, or hangers (in the case of the deck suspended from the arch) that have negligible weight, compared with the weight of the deck and the arch.

As shown in figure 1*b*, the arch is pin-ended, with the vertical reactions at the supports denoted by *V_L_* and *V_R_* respectively, and the equal and opposite horizontal reactions by *H*. An arbitrary point between *O* and *P* has coordinates (*ξ*,*η*), and arch length, ***σ***, measured from *O*. In general, at a given point *P*, the internal forces acting on the arch cross section are: *T*, axial or thrust/compressive force; *S*, shear force and *M*, bending moment.

The equations of equilibrium for the segment *OP* are as follows.

Vertical equilibrium:

2.1 $$-T\sin\theta +S\cos\theta -wx-\int_{0}^{s}qA\;\text{d}\sigma +V_{L}=0.$$

Horizontal equilibrium:

2.2 −Tcos⁡θ−Ssin⁡θ+H=0.

Rotational equilibrium about *P*:

2.3 $$M+Hy-V_{L}x+\frac{wx^{2}}{2}+\int_{0}^{s}qA(x-\xi )\;\text{d}\sigma =0.$$

The equations for *S* and *T* are, therefore:

$$\left[\begin{array}{cc} \cos\theta & -\sin\theta \\ \sin\theta & \cos\theta \end{array}\right]\left[\begin{array}{c} S\\ T \end{array}\right]=\left[\begin{array}{c} wx+\int_{0}^{s}qA\;\text{d}\sigma -V_{L}\\ H \end{array}\right].$$

Solving for *S* and *T* gives:

2.4 $$S=\cos\theta \left(wx+\int_{0}^{s}qA\;\text{d}\sigma -V_{L}\right)+H\sin\theta$$

and

2.5 $$T=-\sin\theta \left(wx+\int_{0}^{s}qA\;\text{d}\sigma -V_{L}\right)+H\cos\theta .$$

Differentiating equation (2.3) with respect to *s*, and combining it with the expression for *S* from (2.4), leads to:

2.6 $$\frac{\text{d}M}{\text{d}s}+S=0.$$

This equation of constraint between *M* and *S* has the important consequence that, in a pin-ended arch, the vanishing of shear, *S*, everywhere, is a necessary and sufficient condition for the arch to be moment-less.

### (b) Governing differential equation

It can be shown (Appendix A) that the governing nonlinear differential equation for the arch centre-line profile, referred to as the shape equation, is

2.7 $$y^{\prime \prime }=-\left[\frac{w}{H}+\frac{q}{f}(1+{y^{\prime }}^{2})\right],$$

with the following conditions:

at *x*  = 0, *y* = 0; at *x* = *l*, *y* = Δ; at *x *= *x_a_*, *y *= *h* and *y′ *= 0.

### (c) The solution of the governing shape equation

The derivation of the solution to equation (2.7), given in Appendix A, provides the centre-line profile of the arch, as follows:

2.8 $$y=h+\frac{1}{\mu }\ln\cos\left(\sqrt{\mu \kappa }(x-x_{a})\right),$$

in which,

2.9 $$\mu =\frac{q}{f},$$

and

2.10 $$\kappa =\frac{w}{H}+\mu .$$

The solution presented above relied on *y′* > 0. It can be readily demonstrated that taking *y′* < 0 leads to the same answer. The parameters *µ* and *κ*, which arise naturally in the derivation of equation (2.8), reveal the dependence of the centre-line profile on loading, which is the essence of the form-finding approach in general.

An equivalent form of equation (2.8), but using different parametrization and origin, can be found in [17], in relation to least weight structures. However, the centre-line equation alone does not provide a complete geometric description of the arch, which also requires expressions for the offset *x_a_*, and the varying cross-section area, *A*, and these are not given in [17]. As shown in §2d below, the offset, *x_a_*, is a function of *µ*, *h* and Δ (figure 1).

### (d) Determination of the unknown parameters and the solution process

In order to determine the centre-line profile of the arch, it is necessary to find the horizontal reaction, *H*, in terms of the independent input variables (*l*, *h*, *q*, *w* and *f*), as well as the horizontal distance to the apex of the arch, *x_a_*. Since *H* enters the equation for the centre-line profile of the arch, its value can be found from the end condition, as follows:

At *x *= 0, *y *= 0:

$$0=h+\frac{1}{\mu }\ln\cos\left(\sqrt{\mu \kappa }(0-x_{a})\right),$$

and

2.11 $$\cos\left(\sqrt{\mu \kappa }x_{a}\right)={\text{e}}^{-\mu h},$$

giving:

$$\kappa =\frac{1}{\mu {x_{a}}^{2}}{\left[\cos^{-1}({\text{e}}^{-\mu h})\right]}^{2}.$$

Consequently, the horizontal reaction is

2.12 $$H=\frac{fqw}{{\left[(\;f/x_{a})\cos^{-1}({\text{e}}^{-\mu h}\right)]}^{2}-q^{2}}.$$

On using the condition *x* = *l*, *y* = Δ, the procedure described above can be repeated to give:

2.13 $$H=\frac{fqw}{{\left[\;f/(l-x_{a})\cos^{-1}({\text{e}}^{-\mu (h-\Delta )}\right)]}^{2}-q^{2}}.$$

Equating the right-hand sides of equations (2.12) and (2.13) leads to:

2.14 $$x_{a}=\frac{\cos^{-1}({\text{e}}^{-\mu h})}{\cos^{-1}({\text{e}}^{-\mu (h-\Delta )})+\cos^{-1}({\text{e}}^{-\mu h})}l.$$

The geometry of the arch is not complete without a knowledge of the distribution of the material along the arch length, described by *A*_0_ and *A*. From horizontal equilibrium, *H*/*f*  = *A*_0_, and substituting this expression to equation (2.12) gives the cross-section area at the apex:

2.15 $$A_{0}=\frac{qw}{{\left[(\;f/x_{a})\cos^{-1}({\text{e}}^{-\mu h})\right]}^{2}-q^{2}}.$$

The parameter *κ* in equation (2.10) can be written as

$$\kappa =\frac{q}{f}\left(1+\frac{w/q}{A_{0}}\right)=\mu \left(1+\frac{w/q}{A_{0}}\right).$$

Inserting the above expression for *κ* into equation (2.8) and substituting for *A*_0_, gives the following form of that equation:

2.16 $$y=h+\frac{1}{\mu }\ln\cos\left(\sqrt{\mu^{2}\left(1+\frac{w/q}{A_{0}}\right)}(x-x_{a})\right)=h+\frac{1}{\mu }\ln\cos\left[\frac{1}{x_{a}}\cos^{-1}({\text{e}}^{-\mu h}(x-x_{a}))\right],$$

from which it can be seen that the centre-line profile is independent of the deck weight, *w*, as previously stated in [16].

The varying cross section of the arch, *A*, is given as

2.17 $$A=\frac{A_{0}}{\cos\theta }=\sqrt{1+y^{\prime 2}}A_{0}=A_{0}\sqrt{1+\frac{\kappa }{\mu }\tan^{2}\left[\sqrt{\mu \kappa }(x-x_{a})\right]},$$

and the volume of the arch, *V*, is determined as follows:

2.18 $$\begin{array}{rl} V & \;=\int_{0}^{C}A\text{d}s=A_{0}\int_{0}^{l}\left[1+\frac{\kappa }{\mu }\text{ta}{\text{n}}^{2}\left(\sqrt{\mu \kappa }(x-x_{a})\right)\right]\text{d}x\\ & \;=A_{0}l+\frac{\kappa }{\mu }A_{0}\int_{0}^{l}\left[\text{se}{\text{c}}^{2}\left(\sqrt{\mu \kappa }(x-x_{a})\right)-1\right]\text{d}x, \end{array}$$

which reduces to:

2.19 $$V=\left(1-\frac{\kappa }{\mu }\right)A_{0}l+\frac{\kappa }{\mu \sqrt{\mu \kappa }}A_{0}\left[\tan\left(\sqrt{\mu \kappa }(l-x_{a})\right)+\tan\sqrt{\mu \kappa }x_{a}\right].$$

The solution process requires a suitable value of constant stress, *f*, to be selected, such that stresses in the structure arising from the ultimate (permanent + live) load do not exceed the design strength of the material. For a chosen material, the volumetric weight density, *q*, is known and, therefore, *µ* *=* *q*/*f* is known. Since the rise, *h*, is also an independent input variable, the horizontal distance to the apex, *x_a_*, can be determined using equation (2.14). The horizontal reaction, *H*, can then be found from equation (2.12) or (2.13), and *κ* from (2.10). Finally, equation (2.8) can then be used to find the unknown vertical coordinates, *y*, of the centre-line profile of the arch. The geometric description of the arch is not complete without defining its cross-section area at the apex, *A*_0_, and the varied cross-section area, *A*; these can be found from equations (2.15) and (2.17), respectively.

It should be noted that the relationship for *A*_0_ given by equation (2.15), as well as equations (2.12) and (2.13) describing *H*, do not hold for the case *w* = 0, i.e. a stand-alone arch carrying its own weight only. In this case, the cross-section area at the apex, *A*_0_, becomes an independent input variable, and the centre-line profile is independent of both *w* and *H*. Consequently, a stand-alone arch requires a separate treatment, as discussed in the next section.

## 3. Asymmetric form of a constant stress arch carrying the arch weight only

From equation (2.8) we have:

$$y=h+\frac{1}{\mu }\text{ln}\cos\left(\sqrt{\mu \kappa }(x-x_{a})\right),$$

with *µ* = *q*/*f* and *κ* = *w*/*H* + *µ*, defined earlier.

In the case when the arch weight is the only load acting on the structure, *w* = 0, and *κ* = *µ*.

Consequently, the equation defining the centre-line profile of the arch is

3.1 $$y=h+\frac{1}{\mu }\text{ln cos}[\mu (x-x_{a})],$$

with *x_a_* defined in equation (2.14).

The above equation shows that the horizontal reaction, *H*, plays no role in describing the centre-line profile of the arch, and the cross-section area at the apex, *A*_0_, becomes an independent input variable defining the size of the arch, and hence the load, acting on the structure. With *A*_0_ assumed, the horizontal reaction is

3.2 $$H=A_{0}\;f,$$

and the cross-section area varying along the span is

3.3 $$A=\frac{A_{0}}{\cos\theta }=\sqrt{1+{y^{\prime }}^{2}}A_{0}=A_{0}\sqrt{1+\text{ta}{\text{n}}^{2}(\mu (x-x_{a})).}$$

With *w* = 0, *κ* = *µ*, and using equation (2.18), the volume of an asymmetric constant stress arch subjected to arch weight only becomes:

3.4 $$V=\frac{1}{\mu }A_{0}[\tan(\mu (l-x_{a}))+\tan\mu x_{a}].$$

## 4. Symmetric form of a constant stress arch subjected to permanent load (arch self-weight and deck weight)

### (a) Centre-line profile and the determination of the unknown, dependent parameters

The arch becomes symmetric, when *x_a_* = *l*/2 and Δ = 0. With the origin moved to the centre of the arch, equation (2.8) gives the centre-line profile for a symmetric arch of constant axial stress, as

4.1 $$y=h+\frac{1}{\mu }\ln\cos\left(\sqrt{\mu \kappa }x\right).$$

Since $\cos\left(\sqrt{\mu \kappa }x\right)<1,$ $\text{ln}\cos\left(\sqrt{\mu }\kappa x\right)<0.$

It was stated earlier, in relation to the asymmetric arch, that the centre-line profile is independent of the deck weight per unit scan, *w*, and the same applies here.

Substituting *x_a_ *= *l*/2 in equation (2.16) gives:

4.2 $$y=h+\frac{1}{\mu }\ln\cos\left[\frac{2}{l}\cos^{-1}({\text{e}}^{-\mu h}x)\right].$$

Using the condition at the pin: at *x *= *l*/2, *y *= 0, equation (4.1) gives:

$$0=h+\frac{1}{\mu }\text{ln}\cos\left(\sqrt{\mu \kappa }\frac{l}{2}\right),$$

and the constraint condition given previously by equation (2.11) becomes:

4.3 $$\cos\left(\sqrt{\mu \kappa }\frac{l}{2}\right)={\text{e}}^{-\mu h}.$$

With *x_a_* = *l*/2, equations (2.12) and (2.13) describing the horizontal reaction, *H*, collapse to just one equation:

4.4 $$H=\frac{fqw}{{\left[(2f/l)\cos^{-1}({\text{e}}^{-\mu h}\right)]}^{2}-q^{2}}.$$

Equation (2.15) describing the cross-section area at the apex now becomes:

4.5 $$A_{0}=\frac{H}{f}=\frac{qw}{{\left[(2f/l)\cos^{-1}({\text{e}}^{-\mu h}\right)]}^{2}-q^{2}},$$

and, with reference to equation (2.17) gives the varying cross-section area:

4.6 $$A=A_{0}\sqrt{1+\frac{\kappa }{\mu }\text{ta}{\text{n}}^{2}\left(\sqrt{\mu \kappa }x\right).}$$

Using equation (2.19), and noting that *x_a_* = *l*/2, the volume of the symmetric form of a constant stress arch subjected to the arch and deck weight is

4.7 $$V=2A_{0}\left[\left(1-\frac{\kappa }{\mu }\right)\frac{l}{2}+\sqrt{\frac{\kappa }{\mu^{3}}}\tan\left(\sqrt{\mu \kappa }\frac{l}{2}\right)\right].$$

### (b) Constraint conditions determining the existence of the constant stress arch

Introducing, for compactness:

$$z=\cos^{-1}({\text{e}}^{-2\gamma /\rho }),$$

where

$$\rho =\frac{l}{h},$$

and

$$\gamma =\frac{ql}{f},$$

equation (4.4) can be rewritten as

4.8 $$H=\frac{wf}{q{\left[(1/\gamma )\cos^{-1}({\text{e}}^{-2\gamma /\rho }\right)]}^{2}-1}=\frac{rf}{{\left(z/\gamma \right)}^{2}-1},$$

where *r* = *w*/*q*.

Consequently:

4.9 $$A_{0}=\frac{H}{f}=\frac{r}{{\left(z/\gamma \right)}^{2}-1}.$$

It is shown in [4] that the ratio of deck to arch densities, *r*, plays a key role in shaping moment-less arches of constant cross section. It can be seen here that it also plays a key role in shaping arches of constant axial stress, as it affects the horizontal reaction *H*, the cross-section area at the apex, *A*_0_ (and, consequently, the varied cross section, *A*), as well as the arch volume, *V*, discussed in §4c.

It can be seen from equation (4.8) that the horizontal reaction, *H*, derived from the end condition, can become negative, when:

4.10 $${\left(\frac{z}{\gamma }\right)}^{2}<1,$$

and *H →* ∞, when

4.11 $$\frac{z}{\gamma }=1.$$

Similarly, from equation (4.9), *A*_0_ < 0 when inequality (4.10) holds, and *A*_0_ → ∞ when the equation (4.11) is satisfied. Equation (4.11) may be rewritten as

$${\text{e}}^{-2\gamma /\rho }=\cos\gamma ,$$

leading to a critical relationship between *ρ* and *γ*, given by

4.12 $$\rho =\frac{2\gamma }{\text{ln}\sec\gamma }.$$

When the above relationship holds, *A*_0_ becomes unbounded, as does the horizontal reaction, *H*. Equation (4.12) also indicates that lnsec⁡γ must not become infinitely large, and for this to happen, the following inequality must be satisfied:

4.13 $$\gamma =\frac{ql}{2f}<\frac{\pi }{2}.$$

**Figure 2.**
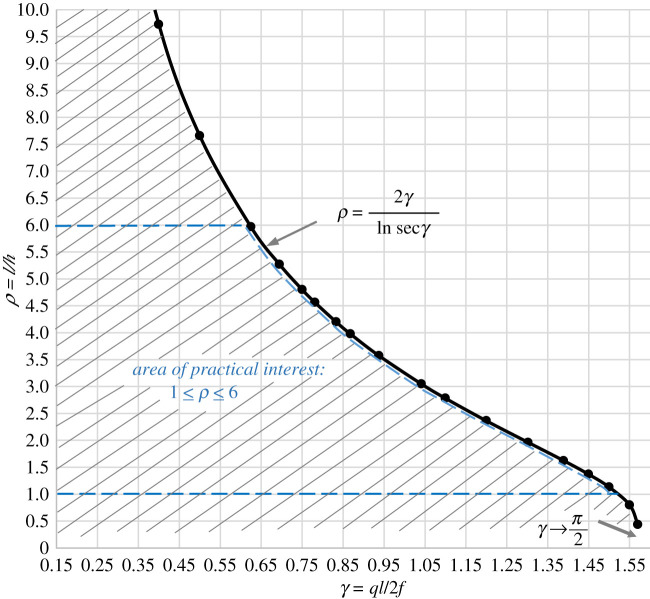
Limiting *ρ*–*γ* relationship defining the design space/existence of a constant stress arch. The hatched area under the curve represents the design space for a fully loaded arch; this area collapses to the *ρ *= 2*γ*/ln sec *γ* curve, for a stand-alone arch (§5). (Online version in colour.)

The condition given in (4.13) has been given in [16] in relation to ‘fully stressed’ least weight arches, but equation (4.12) is a new result, the significance of which merits further analysis. It is shown (Appendix B) that when the span/rise ratio, *ρ*, falls outside the *ρ*—*γ* curve, i.e.

$$\rho >\frac{2\gamma }{\text{ln}\sec\gamma },$$

both the horizontal reaction, *H*, and the cross-section area at the apex, *A*_0,_ become negative (equations (4.8) and (4.9), respectively). At this point, it is helpful to introduce the concept of a *Design Space*, bounded by the critical *ρ* − *γ* curve, as well as the limits on *ρ* and *γ*, as shown in figure 2*.* The design space, which is the area underneath the curve, determines allowable *ρ*, *γ* combinations. For the (*ρ*, *γ*) points lying on the constraint curve, the horizontal reaction, *H*, and the cross-section area at the apex, *A*_0_, become infinitely large; for the (*ρ*, *γ*) points above the curve, they acquire negative values. Thus, the design space limits the existence of a constant stress arch.

With reference to figure 2, it can be seen that, when *γ *→* π*/2, *ρ* → 0, which is of no practical interest, as is the case of *γ* = 0, which, for non-zero values of *l* and *q*, would give: *f* → ∞. Using practical values of the span/rise ratio, between, say, 1 and 6, it is possible to define practical limits of *γ*, using the defined design space limits. It can be seen that *ρ *= 1 gives a number of possible values for *γ*, within the interval 0 < *γ* < *γ*_lim_, where *γ*_lim _≅ 1.5, with a more exact value found from equation (4.12): *γ*_lim_ = 1.52325. When *ρ* = 6, the interval for *γ* is 0 *<* *γ* < *γ*_lim_ ≅ 0.6, with a more exact value: *γ*_lim_ = 0.62189 obtained from equation (4.12). Illustrative calculations of the *ρ*–*γ* limits, related to specific spans for both concrete and steel structures, are given in electronic supplementary material, S1. It is shown in §5 that the design space collapses to the *ρ* = 2*γ*/ln sec *γ* curve in the case of a stand-alone arch.

As stated in §1, there exists an interesting parallel between constant stress arches and (minimal) constant stress surfaces; more specifically, stable minimal surfaces, exemplified by soap films [20,21] and used in conceptual design of fabric structures [3]. Both types of structures are subject to conditions limiting their existence. For example, it is known that a catenoid surface—a surface formed between two co-axial rings (one of the few minimal surfaces that have a closed-form solution)—cannot be formed when the ratio of the ring separation to their radius is greater than 1.325. Of course, infinitely many catenoids, of different ring separations and radii, satisfying the stated limit, can be formed. Similarly, the design space for constant stress arches, although limited, is large enough to accommodate an enormous number of *ρ*–*γ* combinations.

### (c) Span/rise ratio minimizing the volume of material

Equation (4.7) can be reformulated in terms of *r* and *z*, as follows:

Noting that:

$$\begin{array}{rl} H & \;=\frac{fr}{{\left(z/\gamma \right)}^{2}-1},\\ \kappa & \;=\frac{w}{H}+\frac{q}{f}=\frac{q}{f}\left[\frac{wf}{qH}+1\right]=\frac{2\gamma }{l}\left[rf\cdot \frac{{\left(z/\gamma \right)}^{2}-1}{rf}+1\right]=\frac{2\gamma }{l}{\left(\frac{z}{\gamma }\right)}^{2},\\ \mu & \;=\frac{2\gamma }{l},\\ \frac{\kappa }{\mu } & \;={\left(\frac{z}{\gamma }\right)}^{2},\\ \;\text{and}\;\sqrt{\mu \kappa } & \;=\sqrt{\frac{2\gamma }{l}\cdot \frac{2\gamma }{l}{\left(\frac{z}{\gamma }\right)}^{2}}=\frac{2z}{l}, \end{array}$$

gives the following form of equation (4.7):

4.14 $$\begin{array}{rl} V & \;=2A_{0}\left[\left(1-\frac{\kappa }{\mu }\right)\frac{l}{2}+\sqrt{\frac{\kappa }{\mu^{3}}}\tan\left(\sqrt{\mu \kappa }\frac{l}{2}\right)\right]\\ & \;=A_{0}\left\{\left[1-{\left(\frac{z}{\gamma }\right)}^{2}\right]\frac{l}{2}+\sqrt{\frac{2z^{2}}{\gamma l}\cdot {\left(\frac{l}{2\gamma }\right)}^{3}\tan z}\right\}. \end{array}$$

The above simplifies to:

$$V=rl\left[-1+\frac{z}{z^{2}-\gamma^{2}}\tan z\right].$$

Expressing equation (4.14) in non-dimensional form gives:

4.15 $$\bar{V}=\frac{V}{rl}=\left[-1+\frac{z}{z^{2}-\gamma^{2}}\tan z\right].$$

Minimizing $\bar{V}$ with respect to *ρ* gives:

$$\frac{\text{d}\bar{V}}{\text{d}\rho }=\frac{\text{d}\bar{V}}{\text{d}z}\cdot \frac{\text{d}z}{\text{d}\rho }=0.$$

From cos *z *= e^−2^*^γ^*^/^*^ρ^* it follows that:

$$\frac{\text{d}z}{\text{d}\rho }=-\frac{2\gamma {\text{e}}^{-2\gamma /\rho }}{\sqrt{\left(1-{\text{e}}^{-2\gamma /\rho }\right)}}.$$

Consequently:

$$\frac{\text{d}\bar{V}}{\text{d}\rho }=\left[\frac{(z^{2}-\gamma^{2})-2z^{2}}{{\left(z^{2}-\gamma^{2}\right)}^{2}}\tan z+\frac{z}{\left(z^{2}-\gamma^{2}\right)}\sec^{2}z\right]\frac{\text{d}z}{\text{d}\rho }.$$

As d*z*/d*ρ* is non-zero, the equation for volume minimization is

4.16 $$\text{ta}{\text{n}}^{2}z-\frac{z^{2}+\gamma^{2}}{z(z^{2}-\gamma^{2})}\tan z+1=0,$$

and the solution to it, say, $\bar{z}$, can be obtained using a number of numerical methods.

With $\bar{z}$ determined from equation (4.16), the span/rise ratio, *ρ*_min,_ at which the arch becomes a structure of least weight, is

4.17 $$\rho_{\text{min}}=-\frac{2\gamma }{\ln\cos\bar{z}}.$$

Using *ρ*_min_ and chosen input parameters: *l*, and *µ*, the centre-line profile of a least weight arch can be found from equation (4.2), using *h *= *l*/*ρ*_min_.

Results of the volume minimization analysis for an arch made of concrete are summarized in table 1. The full set of results is given in electronic supplementary material, S2. With reference to table 1, it can be seen that, in the case *f* = 1.5 MPa, and *γ* = 1.6667, the span/rise ratio, *ρ*_min_, is not reached, because the condition given by equation (4.13), also shown in figure 2, is violated, i.e. *γ* > *π*/2.

**Table 1.** Values of span/rise ratio, *ρ*_min_ minimizing material volume and $\bar{z}$_._

	*l* = 50 m			*l* = 100 m			*l* = 150 m			*l* = 200 m		
*f*	*γ*	$\bar{z}$	$\rho_{\text{min}}$	*γ*	$\bar{z}$	$\rho_{\text{min}}$	*γ*	$\bar{z}$	$\rho_{\text{min}}$	*γ*	$\bar{z}$	$\rho_{\min}$
(1)	(2)	(3)	(4)	(5)	(6)	(7)	(8)	(9)	(10)	(11)	(12)	(13)
3.6 MPa	0.1736	0.5431	2.23	0.3472	0.7616	2.15	0.5208	0.9257	2.05	0.6944	1.0618	1.93
2.4 MPa	0.2604	0.6623	2.19	0.5208	0.9256	2.05	0.7813	1.1229	1.87	1.0417	1.2873	1.64
1.8 MPa	0.3472	0.7616	2.15	0.6944	1.062	1.93	1.0417	1.2873	1.64	1.3889	1.4782	1.17
1.5 MPa	0.4167	0.8316	2.11	0.8333	1.1578	1.64	1.25	1.4046	1.39	1.6667	not available	not available

**Figure 3.**
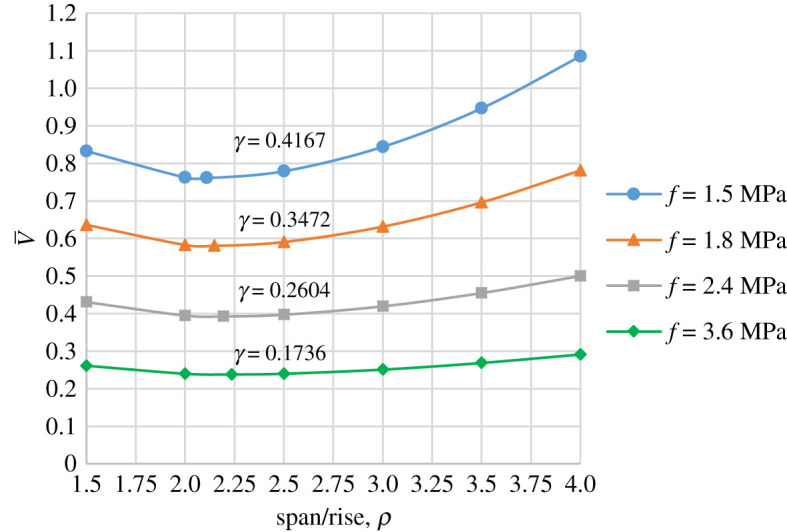
Variation of $\bar{V}$ with *ρ*, for a range of *γ* corresponding to chosen values of constant stress, *f*. Other data: *q* = 25 kN m^−3^, *r* = 2 and span *l* = 50 m. (Online version in colour.)

Using an example of a constant stress arch of span *l* = 50 m, and the following additional input parameters: *w* = 50 kN m^−1^, *q* = 25 kN m^−3^ (corresponding to concrete weight density), the volume of the arch can be calculated from equation (4.7), or (4.14) for a range of span/rise ratios and stress values. The results are presented in figure 3, with detailed calculations given in supplementary electronic material, S2.

With reference to figure 3, it can be seen that, for a given span and stress (producing specific values of *γ*), the span/rise ratio minimizing the volume of the material in the arch, *ρ*_min_ is just above 2. The more exact values for *ρ*_min_ are given in column 4 of table 1.

A similar study can be carried out for other spans, as shown in table 1, and for steel structures, with *q* = 75 kN m^−3^ and a range of stress appropriately scaled up.

Each curve in figure 3 represents a family of constant stress arches that can be generated within a given span, *l*, depending on the chosen span/rise ratio, *ρ*, and value of stress, *f*. It can be checked that this family falls safely into the design space given in figure 2.

**Figure 4.**
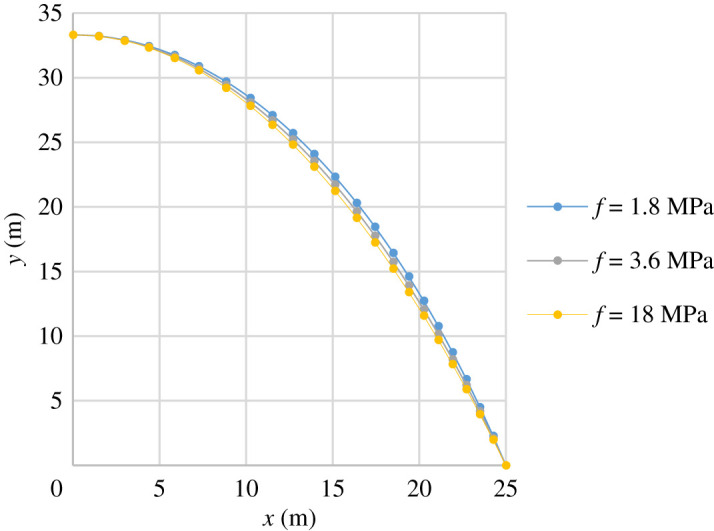
Centre-line profiles of a high-rise arch, for varying values of constant stress, *f*. Other data: *ρ* = 1.5, *q* = 25 kN m^−3^, *r* = 2, *l* = 50 m. (Online version in colour.)

Figure 3 indicates that arch volume is influenced by the value of stress. Considering an arch of span/rise *ρ* = 1.5 and the same input parameters *q*, *r* and *l* as in figure 3, it is possible to examine the effect of stress on the arch profile and, consequently, the volume of material. Using a range of stress values, *f* (corresponding to specific values of *γ*), the centre-line profiles are plotted in figure 4. The results show that stress has only a small effect on the centre-line profile of the arch, and hence, the change in volume is largely due to the cross-section area being affected.

## 5. Symmetric form of a constant stress arch subjected to arch weight only: the stand-alone arch

### (a) Centre-line profile

With the deck weight, *w* = 0, *κ* = *μ*, the centre-line equation (4.1) becomes:

5.1 $$y=h+\frac{1}{\mu }\ln\cos\mu \;x,$$

where, for a bounded solution, μl/2<π/2.

It can be seen from equation (5.1) that the horizontal reaction, *H*, plays no role in determining the centre-line profiles of the arch. Consequently, it is not possible to find a value of *H* that minimizes the arch weight/volume that the structural optimization methodology relies on.

### (b) Remaining design parameters

As stated in §2d, the apex cross section of the arch, *A*_0_, can no longer be determined from the equations given for the fully loaded arch, as it now becomes an independent variable. With *A*_0_ and *f* selected, the horizontal reaction is given by equation (3.2) and the varying cross section, *A*, is

5.2 $$A=\frac{A_{0}}{\cos\theta }=\sqrt{1+{y^{\prime }}^{2}}A_{0}=A_{0}\sqrt{1+\text{ta}{\text{n}}^{2}(\mu x).}$$

The volume of the arch can be found from equation (4.7), which, on substitution *κ* = *μ*, gives:

5.3 $$V=2\frac{A_{0}l}{\mu }\tan\left(\mu \frac{l}{2}\right),$$

or, since *γ *= *ql*/2*f *= *μl*/2

5.4 $$V=\frac{A_{0}l}{\gamma }\tan\gamma .$$

Using a normalized value of *V*, say, $\bar{V}$:

5.5 $$\bar{V}=\frac{V}{A_{0}l}=\frac{1}{\gamma }\tan\gamma .$$

As can be seen from equation (5.4), the volume of a stand-alone arch depends on *γ*. As *γ *→ *π*/2, the volume increases without limit.

### (c) Constraint condition

Implementing the end condition: *x *= *l*/2, *y *= 0, gives

5.6 $$0=h+\frac{1}{\mu }\text{ln}\cos\left(\mu \frac{l}{2}\right),$$

or

$${\text{e}}^{-\mu h}=\cos\left(\mu \frac{l}{2}\right)=\cos\left(\frac{q}{f}\frac{l}{2}\right)=\cos\gamma .$$

which, in terms of *ρ* and *γ*, becomes:

5.7 $${\text{e}}^{-2\gamma /\rho }=\text{cos}\gamma ,$$

or:

5.8 $$\rho =\frac{2\gamma }{\text{ln}\;\text{sec}\;\gamma }.$$

Equation (5.8) is of the same form as equation (4.12), which, in the case of a fully loaded arch, marked an exclusive boundary of the available design space for arbitrarily chosen *ρ* and *γ*. Here, the same equation has a different significance; it shows that, for a given *ρ,* there can be only one value of *γ.* Thus, for a chosen material characterized by its weight density, *q*, and a given span*, l*, there exists a unique relationship between *ρ* and stress, *f*. This reduces the design space to ρ=2γ/ln⁡sec⁡γ curve, shown in figure 2.

As *ρ* is an input parameter, the required value of stress can be found as shown below.

From equation (5.6) we have:

$$0=h\mu +\text{ln}\cos\left(\mu \frac{l}{2}\right).$$

Introducing *u* *=* *μl*/2 and noting that *hμ *= *l*/*ρ* · 2*u*/*l*, we get:

5.9 $$0=\frac{2u}{\rho }+\text{ln cos}u.$$

Solving the above equation for *u*, gives *μ* that can be used to: (i) determine the required value of constant stress, *f* = *q*/*μ*, and (ii) find the *y* coordinates of the arch from equation (5.1). Detailed calculation for *f* is given in the electronic supplementary material, S3.

**Figure 5.**
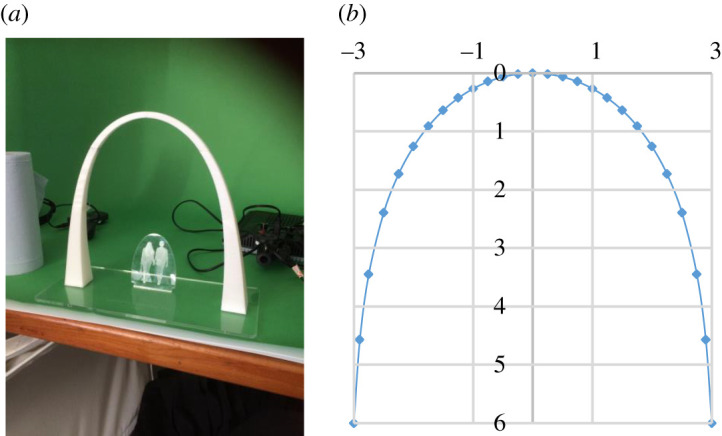
Stand-alone, constant stress arch (*a*) 1 : 20 model and (*b*) arch centre-line profile. Input variables for a real-size structure: *q* = 25 kN m^−3^, *l* = 6 m, *ρ* = 1, *A*_0_ = 0.3 m^2^; output variables: centre-line profile, constant stress, *f*, and variable cross-section area, *A*. The model and image in (*a*) courtesy of M. Millson, University of Warwick. (Online version in colour.)

Figure 5 shows a 1 : 20 model of a small, stand-alone, constant stress arch. The arch is assumed to have a square cross section varying along its length. It is also possible to select a rectangular cross section, with the larger dimension oriented in the transverse direction, to provide greater lateral stability for the arch. The stress, *f*, required to satisfy equation (5.8) is found to be 0.049 MPa, and the arch volume (equation (5.4)) is 2.483 m^3^. It can be checked that, for *ρ* = 1, figure 2 gives *γ *≅ 1.52, from which *f *≅ 0.049 MPa. Detailed calculations of the arch centre-line profile and volume are given in electronic supplementary material, S3.

## 6. Summary and conclusion

This paper presents a model of a smooth two-pin, moment-less arch, shaped by a constant value of axial stress and statistically prevalent (permanent) load, such as the weight of the structure. The proposed methodology represents an advancement in the analytical form-finding approach, previously used in [4] in relation to inextensible moment-less arches of constant cross section, by proposing a constant axial stress criterion that defines moment-less arches of varied cross section. The theory describes a general case of an asymmetric structure, from which a symmetric form is deduced. It covers arches that are fully loaded (carrying the deck and arch weight), and stand-alone (carrying arch weight only).

Important design factors considered in the proposed model are the level of constant stress and the type of load used in shaping the structure. When the stress value corresponds to the ultimate load (comprising permanent and variable load), the solution produces a constant stress response just for this transient condition. This means the structure will develop a varied stress state under permanent load, which impacts its durability. For this reason, the proposed work advocates the use of permanent load to determine the arch geometry. If, under the ultimate load, the structure were to exceed its design strength, key input parameters, such as the value of constant stress, can be changed, and the arch geometry recalculated, as part of a usual design process.

Compared to optimization approaches focusing on least weight structures, the proposed analytical form-finding, defining constant stress arches, opens up design opportunities by offering not one, but a range of span/rise ratios for these natural structural forms, provided they fall into the available design space—a new concept derived here.

In the case of fully loaded arches, the design space comprises combinations of two dimensionless input parameters, *ρ* and *γ*, which can be selected independently, but must fall within the limits determining the existence of the arch.

In the case of stand-alone arches, the design space collapses to the bounding *ρ*–*γ* curve itself, giving a constraint relationship between *ρ* and *γ*, which, for a given span and loading, becomes a constraint relationship between *ρ* and *f—*the value of constant stress.

The proposed design space indicates that the well-known limit *γ* < *π*/2 falls into the area of no practical interest, because, at this point, the span/rise ratio tends to zero. It is shown that limits on *γ*, corresponding to practical span/rise ratios, can be found from the key *ρ − γ* relationship derived in this study.

While not focusing on structures of least weight, the proposed approach allows them to be found, by providing an explicit solution for the span/rise ratio minimizing the volume of a constant stress arch. It is confirmed that the deck weight does not influence the centre-line profile of the arch; instead, it affects its material distribution along the arch length and, consequently, its volume.

Constant stress arches provide efficient and durable design solutions, because of their simple stress response to statistically prevalent loading. Although somewhat restricted, their design space is large enough for them to be exploited in conceptual design.

Further work will examine arch stability, and propose appropriate levels of constant stress, so that the arches do not exceed their design strength limits when subjected to the ultimate load, or become too heavy, when the chosen value of stress is too low.

## Data accessibility

Geometrical and stress analyses data are provided in the electronic supplementary material.

## Appendix A

**A.1. Governing differential equation**

The vertical reaction *V*_L_ can be found by considering rotational equilibrium about the right-hand support (figure 1), from which:

$$V_{L}=\frac{\Delta }{l}H+\frac{wl}{2}+\frac{1}{l}\int_{0}^{C}qA(l-\xi )\;\text{d}\sigma .$$

Substituting *V_L_* into equation (2.4) and re-grouping gives:

$$S=\cos\theta \left[-w\left(\frac{l}{2}-x\right)-\int_{s}^{C}qA\;\text{d}\sigma +\frac{1}{l}\int_{0}^{C}qA\xi \;\text{d}\sigma \right]+\left(\sin\theta -\frac{\Delta }{l}\cos\theta \right)H.$$

Imposing the condition of zero shear everywhere and using tan*θ *= *y*^′^, where *y′* is the first derivative with respect to *x*, gives:

$$Hy^{\prime }=\frac{\Delta }{l}H+w\left(\frac{l}{2}-x\right)+\int_{s}^{C}qA\;\text{d}\sigma -\frac{1}{l}\int_{0}^{C}qA\xi \;\text{d}\sigma .$$

Differentiating with respect to *x* and noting that $\text{d}s/\text{d}x=\sqrt{1+{y^{\prime }}^{2}}$

gives:

$$y^{\prime \prime }=-\left[\frac{w}{H}+\frac{qA}{H}\sqrt{1+{y^{\prime }}^{2}}\right].$$

Since *S* = 0, then from equation (2.2):

$$T=\frac{H}{\cos\theta }$$

and since *T *= *fA*, and $\cos\theta =\sqrt{1+{y^{\prime }}^{2}}$, the cross-section area, *A*, can be eliminated. Consequently, the governing nonlinear differential equation for the arch centre-line profile, known as the *shape equation*, is

$$y^{\prime \prime }=-\left[\frac{w}{H}+\frac{q}{f}(1+{y^{\prime }}^{2})\right],$$

with the boundary conditions: *x* = 0, *y* = 0; *x* = *l*, *y* = Δ; $x=x_{a},y=h\;\text{and}\;y^{\prime }=0$.

**A.2. The solution of the governing shape equation**

The following transformation of *y′′* can be used:

$$y^{\prime \prime }=\frac{\text{d}y^{\prime }}{\text{d}y}\cdot \frac{\text{d}y}{\text{d}x}=y^{\prime }\frac{\text{d}y^{\prime }}{\text{d}y}=\frac{1}{2}\frac{\text{d}}{\text{d}y}(1+{y^{\prime }}^{2}),$$

and the shape equation then becomes:

$$\frac{1}{2}\frac{d}{dy}(1+{y^{\prime }}^{2})=-\left[\frac{w}{H}+\frac{q}{f}(1+{y^{\prime }}^{2})\right].$$

Separating the variables, gives:

$$\frac{f}{2q}\text{ln}\left(\frac{w}{H}+\frac{q}{f}(1+{y^{\prime }}^{2})\right)=-y+k_{1},$$

where *k*_1_ is an arbitrary constant.

At *x* = *x_a_*, *y* = *h*, and *y′* = 0, which gives:

$$k_{1}=h+\frac{f}{2q}\text{ln}\left(\frac{w}{H}+\frac{q}{f}\right).$$

Introducing parameters: *μ *= *q*/*f* and *κ *= *w*/*H *+ *μ*, leads to:

$$\frac{1}{2\mu }\text{ln}\frac{\kappa +\mu {y^{\prime }}^{2}}{\kappa }=h-y,$$

and solving for *y′* gives:

$$y^{\prime }=\pm \sqrt{\frac{\kappa }{\mu }({\text{e}}^{2\mu (h-y)}-1)}.$$

Taking the positive radical and separating the variables gives:

$$\int \frac{\text{d}y}{\sqrt{{\text{e}}^{2\mu (h-y)}}-1}={\left(\frac{\kappa }{\mu }\right)}^{1/2}x+k_{2},$$

where *k*_2_ is an arbitrary constant.

Evaluation of the above integral gives:

$$\int \frac{\text{d}y}{\sqrt{{\text{e}}^{2\mu (h-y)}-1}}=\int \frac{{\text{e}}^{-\mu (h-y)}\;\text{d}y}{\sqrt{1-{\text{e}}^{-2\mu (h-y)}}}=\frac{1}{\mu }\text{si}{\text{n}}^{-1}({\text{e}}^{-\mu (h-y)})=\sqrt{\frac{\kappa }{\mu }}x+k_{2},$$

and applying the condition *y* = *h* at *x* *=* *x_a_* gives:

$$\text{si}{\text{n}}^{-1}(1)=\sqrt{\mu \kappa }x_{a}+k_{2}=\frac{\pi }{2},$$

and

$$k_{2}=\frac{\pi }{2}-\sqrt{\mu \kappa }x_{a}.$$

Consequently, the final solution for the centre-line profile of an asymmetric, constant stress arch is

$$y=h+\frac{1}{\mu }\ln\cos\left(\sqrt{\mu \kappa }(x-x_{a})\right).$$

## Appendix B

Here, it is shown that the horizontal reaction, *H*, and, consequently, the cross-section area at the apex, *A*_0_, become negative, if the span/rise ratio, $\bar{\rho }$, is chosen to lie above the bounding curve in figure 2.

For some *λ *> 1, we choose $\bar{\rho }$ as

$$\bar{\rho }=\frac{2\gamma }{\text{ln}\;\text{sec}\;\gamma }.$$

Then, the denominator in equations (4.8) and (4.9) becomes:

$$\begin{array}{rl} {\left[\frac{1}{\gamma }\cos^{-1}({\text{e}}^{-(2\gamma /\bar{\rho })})\right]}^{2}-1 & \;={\left[\frac{1}{\gamma }\cos^{-1}({\text{e}}^{-(2\gamma /2\gamma \lambda )/\text{lnsec}\gamma })\right]}^{2}-1\\ & \;={\left[\frac{1}{\gamma }\cos^{-1}(e^{-(1/\lambda )\ln\text{sec}\gamma })\right]}^{2}-1={\left[\frac{1}{\gamma }\cos^{-1}({\text{e}}^{\text{ln}{\left(\cos\gamma \right)}^{1/\lambda }})\right]}^{2}-1\\ & \;={\left[\frac{1}{\gamma }\cos^{-1}({\left(\cos\gamma \right)}^{1/\lambda })\right]}^{2}-1. \end{array}$$

Since cos *γ* < 1, and *λ *> 1, ${\left(\cos\gamma \right)}^{1/\lambda }>\cos\gamma$. Choosing $\cos\alpha ={\left(\cos\;\gamma \right)}^{1/\lambda }$, then cos *α *> cos *γ*, and 0 < *α *< *γ*. Consequently,

$${\left[\frac{1}{\gamma }\cos^{-1}({\text{e}}^{-(2\gamma /\bar{\rho })})\right]}^{2}-1={\left[\frac{1}{\gamma }\cos^{-1}(\cos\alpha )\right]}^{2}-1={\left(\frac{\alpha }{\gamma }\right)}^{2}-1<0,$$

and it follows that *H* and *A*_0_ become negative.
